# Factors that protect against poor sleep quality in an adult lifespan sample of non-Hispanic Black and non-Hispanic White adults during COVID-19: A cross-sectional study

**DOI:** 10.3389/fpsyg.2022.949364

**Published:** 2022-12-15

**Authors:** Emily Hokett, Aditi Arunmozhi, Jessica Campbell, Audrey Duarte

**Affiliations:** ^1^Department of Neurology, Columbia University Irving Medical Center, New York, NY, United States; ^2^Milken Institute School of Public Health, George Washington University, Washington, DC, United States; ^3^Department of Psychology, Georgia Institute of Technology, Atlanta, GA, United States; ^4^Department of Psychology, University of Texas at Austin, Austin, TX, United States; ^5^Department of Neurology, University of Texas at Austin, Austin, TX, United States

**Keywords:** sleep quality, race, COVID-19 pandemic, religiosity, social support

## Abstract

**Introduction:**

Stress in relation to the Coronavirus disease 19 pandemic (i.e., COVID-19, COVID stress) may be linked with poor sleep quality. The association between stress that is specific to the COVID-19 pandemic and sleep quality has been understudied, particularly in racially diverse people across the adult lifespan. Here, we investigated self-reported sleep quality in relation to COVID stress and factors that may protect against experiencing poor sleep quality from high COVID stress, including social support and religiosity.

**Method:**

We recruited non-Hispanic Black (*n* = 73) and non-Hispanic White (*n* = 178) participants across the adult lifespan (18–76 years) using an online, cross-sectional design during the COVID-19 pandemic (March 2021–June 2021). We asked participants to report information regarding demographics (age, race/ethnicity, years of education), sleep (sleep quality, sleep habits), and positive (social support, religious activities) and negative (events of discrimination, depression, general stress, COVID stress) psychosocial factors.

**Results:**

Across age and racial groups, better sleep habits were associated with better sleep quality, and higher COVID stress was linked to poorer sleep quality. Black participants reported higher quality sleep than White participants (*p* = 0.006). They also endorsed greater private and internal religiosity (*p’s* < 0.001). Across racial groups, moderation analyses revealed a protective effect of religiosity against poor sleep (*p’s* < 0.006). Specifically, individuals with high religious activity and high COVID stress did not experience poor sleep quality, but individuals with low religious activity and high COVID stress demonstrated poor sleep quality. These results remained significant when controlling for general stress.

**Discussion:**

Protective factors, such as religiosity, may mitigate the negative associations between high COVID stress and poor sleep quality.

## 1 Introduction

There are clear racial disparities in sleep quality within the current body of sleep literature. Non-Hispanic Black adults often sleep more poorly than non-Hispanic White adults (hereafter referred to as Black and White; for a review, Johnson et al., 2019). This racial sleep disparity has been detected using self-report, actigraphy, and polysomnography-measured sleep quality (Tomfohr et al., 2012; Turner et al., 2016; Hokett and Duarte, 2019). In parallel with poorer sleep quality, racial minorities tend to have poorer health outcomes, including higher rates of cardiovascular disease and dementia as compared to White adults (Carnethon et al., 2017; Mehta and Yeo, 2017). Longitudinal studies have shown that high quality sleep at baseline is linked with lower risk of cognitive decline and better cardiovascular health (Chaput et al., 2020; Xu et al., 2020). This suggests that identifying factors that may be detrimental to sleep quality and those that are protective against the negative effects on sleep from those factors may be impactful for maintaining general health and thus narrowing health disparities.

Two factors that may negatively impact poor sleep are stress and discrimination, both of which disproportionately affect Black adults as compared to White adults (Slopen et al., 2016; Williams, 2018). In fact, some evidence suggests that racial sleep disparities may be partially explained by higher levels of discrimination and race-related stress (Slopen and Williams, 2014). A recent study in young adults demonstrated that stress related to the Coronavirus disease 19 pandemic (e.g., essential worker status; hereafter referred to as COVID-19) partially explained poorer self-reported sleep quality in Black adults as compared to other racial/ethnic groups (Yip et al., 2021). While there is some research assessing factors that contribute to poor sleep quality (e.g., stress; for a review, Lo Martire et al., 2020), there is much less research on identifying factors that are related to better sleep, such as self-care (Werner et al., 2021), psychological wellbeing (Tousignant et al., 2022), religiosity (for a review, Hill et al., 2018), and social support (for a review, Kent de Grey et al., 2018) that may protect against the negative impact of stress on sleep quality in adults (Pow et al., 2017). The research on positive factors that are linked to better sleep is particularly understudied in racially diverse adults. The present study aims to address these limitations by focusing on positive (related to better sleep quality) and negative (related to worse sleep quality) sleep co-factors in a sample of Black and White people across the adult lifespan.

There are several factors that may be positively associated with better sleep quality, including educational attainment, sleep habits, social support, and religiosity. Higher education levels have been linked with better self-reported sleep quality (Turner et al., 2016) and better sleep habits (Nam et al., 2018). Poor sleep habits can be characterized as behaviors that are disruptive to sleep. For example, poor sleep habits include using the bed for reasons other than sleep (e.g., watching television, planning, worrying, eating) and engaging in mentally or physically stressful behaviors prior to going to bed (e.g., paying bills or intensely exercising). Better sleep habits have consistently been linked with higher quality sleep and lower self-reported stress in both young and older adults (Ayoub et al., 2014; Anwer et al., 2019). Social support and religious activities may protect against experiencing stressful thoughts and physical discomforts (e.g., muscle tension) before sleep, thus allowing for high quality sleep (Calvete and Connor-Smith, 2006; for a review, Morin et al., 2003; Hill et al., 2018). Furthermore, those who report higher perceived social support are more likely to engage in positive reframing than those who report lower social support (Calvete and Connor-Smith, 2006). The cognitive restructuring facilitated by high social interaction may help to minimize stressful thoughts before sleep. Religious activity may be more directly related with low stress, as researchers have posited that religious activities could deter individuals from risky, stress-inducing behaviors (e.g., criminal behavior, infidelity; for a review, Hill et al., 2018). The current research demonstrates that positive lifestyle and psychosocial factors, such as high educational attainment, social support, religiosity, and appropriate sleep habits may facilitate high sleep quality. However, the protective potential of positive factors against the negative effects of stress on sleep quality is not well understood and understudied in racially diverse adults.

Sleep problems during COVID-19 have been reviewed (Alimoradi et al., 2021; Jahrami et al., 2021). Mental health problems, including severe depression, anxiety, and stress have been associated with higher sleep difficulties (e.g., trouble with sleep initiation and maintenance) during the COVID-19 pandemic (Franceschini et al., 2020). There is much less research on positive factors that may counteract sleep problems due to COVID-19 (e.g., wellbeing, self-care, problem-focused coping; Tracy et al., 2021; Werner et al., 2021; Tousignant et al., 2022). The research that does investigate factors that may positively relate to sleep quality during COVID-19 either underrecruits Black adults (Tracy et al., 2021; Tousignant et al., 2022) or does not report race/ethnicity (Werner et al., 2021). One study found that greater religious experiences were associated with lower perceived stress during the pandemic in Malaysian adults (Ting et al., 2021). Greater positive factors, like religion, may attenuate the association between high stress and poor sleep.

We hypothesize that better sleep habits, higher social support, and greater religiosity will be linked with better sleep quality. Moreover, we expect positive factors, high social support, and religiosity, to moderate associations between high stress and poor sleep quality, with those who are high on these positive factors showing weaker associations between COVID stress and poorer sleep quality than those who are low on positive factors. To test the generalizability of our findings across race/ethnicity, we explore racial group as a moderator for associations between positive and negative factors and sleep quality.

## 2 Materials and methods

We recruited non-Hispanic Black and non-Hispanic White people across the adult lifespan (hereafter referred to as Black and White) using an online crowdsourcing recruitment service, Prolific.co, during COVID-19 (March 2021–June 2021). Researchers and research participants across the globe currently use Prolific, a UK-based company, for research. Prolific allows researchers to advertise their studies to anonymous participants from Prolific’s subject database according to demographics of interest. Participants were required to be U.S. residents, between 18 and 80 years of age, have sufficient eyesight (e.g., ability to clearly see a computer screen), and have proficiency in the English language. They were paid $20 for completing the experiment. Consent forms were approved by the Georgia Institute of Technology Institutional Review Board. All participants completed consent forms before starting the study.

Participants were asked to complete a series of questionnaires using Qualtrics (see Section “2.1 Measures” below for a description of each questionnaire). Qualtrics, a company based in the United States, is a commonly used survey-based platform designed for researchers.^1^ The questionnaires were completed in two separate sessions, spaced 48 h apart, to avoid participant fatigue, as the questionnaire data was collected in addition to a memory study (data not presented here). Each session lasted approximately 1 h.

### 2.1 Measures

Each of the questionnaires described below can be found in Supplementary appendix A. Summaries of these measures are provided below.

#### 2.1.1 Demographics and general health

We collected basic demographic information regarding age, gender, race, ethnicity, years of formal education, and education quality [self-reported measure ranging from 0 (poor) to 2 (excellent)]. To measure income to needs, we assessed financial strain with a single question, “Overall, which one of the following best describes how well you are managing financially these days?” (Szanton et al., 2010). Responses included “living comfortably,” “doing okay,” “just getting by,” and “finding it difficult to get by.” We also developed items to assess general health (e.g., self-reported hypertension, neurological disease, mental illness).

#### 2.1.2 Sleep

We measured sleep quality over the past month with the Pittsburgh Sleep Quality Index (PSQI; Buysse et al., 1989). Using the PSQI, we computed the standard, global measure of sleep quality. Higher scores represent poorer sleep quality (range: 0–21). Participants also estimated their sleep duration using the PSQI.

#### 2.1.3 Positive and negative factors in relation to sleep quality

We measured several positive and negative factors that may be predictive of sleep quality with questionnaires (see Supplementary appendix A). Positive factors included good sleep habits, high social support, and high religiosity. The negative factors included several domains of psychosocial stressors, including general stress, race-related stress, and our primary stress measure involved stress that was specifically related to COVID-19 (i.e., COVID stress).

##### 2.1.3.1 Positive factors

###### 2.1.3.1.1 Sleep habits

We measured sleep habits using the Sleep Hygiene Index (SHI; Mastin et al., 2006). Greater endorsement of behaviors that were not conducive to high quality sleep (e.g., watching television while in bed) is indicative of poorer sleep habits (range: 0–52). Higher scores reflect poorer sleep habits.

###### 2.1.3.1.2 Social support

To assess social support, we examined social support measures that may counteract stress and facilitate better sleep quality, including the degree of emotional support and positive social interaction (Morin et al., 2003; Calvete and Connor-Smith, 2006) using subscales from the Medical Outcomes Study (MOS) social support survey [MOS; (range: 1–5); Sherbourne and Stewart, 1991]. Higher scores are representative of more social support.

###### 2.1.3.1.3 Religiosity

To isolate social support from religiosity, we assessed the frequency of private religious activities (range: 1–6) and degree of internal religiosity (range: 3–15) with The Duke Religion Index (DUREL; Koenig and Büssing, 2010). Higher scores indicate higher religiosity.

##### 2.1.3.2 Negative factors

###### 2.1.3.2.1 Stress, anxiety, and depression

We measured general stress (range: 0–34) using the depression, anxiety, and stress scale-21 (DASS-21; Lovibond and Lovibond, 1995). We developed a COVID stress measure to assess the degree of strain experienced from emotional, financial, and social stressors associated with COVID-19. The COVID stress measure was comprised of five items. Responses ranged from 0 (did not experience) to 3 (high strain). Higher scores were indicative of greater COVID stress (range: 0–12). The internal consistency for the COVID stress, measured with the Spearman-Brown Formula, is 0.75.

###### 2.1.3.2.2 Discrimination

To assess race-related stress, we measured the number of events (range: 0–9) and frequency (range: 0–45) of discrimination with the events of discrimination scale (EOD; Krieger et al., 2005). More events represent more distinct situations of discrimination. More encounters of the events represent higher frequency of discrimination.

### 2.2 Covariates

We wanted to ensure that racial differences in measures that have been linked to poor sleep did not confound our analyses. To this end, we included age, years of education, and education quality as covariates in our statistical models.

### 2.3 Data analysis

Statistical analyses were conducted using the statistical package of social sciences 27 (SPSS). In each analysis, we controlled for covariates as appropriate. First, we examined racial group differences in demographics, sleep, and psychosocial factors using independent *t*-tests and analysis of covariance (ANCOVA). Second, across age and racial groups, we assessed if psychosocial and lifestyle factors were linked with better sleep quality using multiple linear regression models for each factor. Next, we determined if there were racial group differences in associations between psychosocial and lifestyle factors and sleep quality. For these analyses, we employed the PROCESS macro in SPSS. Briefly, we assessed the additional influence of the interaction between racial group and each given factor on global sleep quality, while controlling for covariates. We followed any significant, categorical moderation effects with Pearson’s correlations for each racial group. Lastly, we employed moderation analyses across racial group to examine if positive factors (e.g., social support, religiosity) protect against factors that may be negatively related to sleep quality (e.g., COVID stress). We followed significant, continuous moderation effects with simple slopes at three points–the mean and one standard deviation (SD) below and above the mean. Statistical significance for this study was set to an alpha level of 0.05.

## 3 Results

There were 364 participants who completed both sessions. Of the 364 participants, we excluded 71 with incomplete questionnaire data, 29 who self-reported neurological disease, and 13 who identified as Hispanic/Latino. Thus, our analytical sample includes 251 participants with complete data.

### 3.1 Racial group differences in demographics, psychosocial factors, and sleep

We first assessed racial group differences in demographics, psychosocial factors, and sleep. Black adults were significantly younger than White adults [*t*(189.42) = 3.56, *p* < 0.001]. Thus, age was included as a covariate when assessing racial group differences. Black adults reported greater years of education [*F*(1, 248) = 12.85, *p* < 0.001, η*p2* = 0.049] but lower education quality [*F*(1, 248) = 6.09, *p* = 0.014, η*p2* = 0.024]. Black adults also reported more experiences of discrimination [*F*(1, 248) = 185.83, *p* < 0.001, η*p2* = 0.428] and higher frequency [*F*(1, 248) = 148.44, *p* < 0.001, η*p2* = 0.374] of discrimination than White adults. Black adults endorsed high religiosity [private: *F*(1, 248) = 16.40, *p* < 0.001, η*p2* = 0.062; internal: *F*(1, 248) = 31.81, *p* < 0.001, η*p2* = 0.114], lower depression [*F*(1, 248) = 10.93, *p* = 0.001, η*p2* = 0.042], and better sleep quality [*F*(1, 248) = 7.72, *p* = 0.006, η*p2* = 0.030] as compared to White adults. There were no other significant differences between the racial groups (*p’s* > 0.078). See Table 1 for a summary of descriptive statistics by racial group. See Supplementary appendix B for a histogram of sleep quality for each racial group.

**TABLE 1:** Participant demographics by racial group.

	Black (*n* = 73)	White (*n* = 178)	Significant
Sex (female)	26 (47)	70 (107)	
Age	36.62 (11.46)	43.08 (16.4)	*
Education years	16.7 (2.26)	15.56 (2.37)	*
Education quality	0.85 (0.4)	1.09 (0.68)	*
Financial strain	2.10 (0.78)	2.14 (0.90)	
Sleep quality	5.63 (3.71)	7.01 (3.89)	*
Sleep quality >5	54.3%	69.7%	*
Sleep duration (hours)	6.83 (1.44)	6.98 (6.09)	
Sleep habits	18.92 (8.73)	18.41 (7.67)	
Positive social interaction	3.83 (1.04)	3.85 (1.07)	
Emotional social support	3.88 (0.96)	3.79 (1)	
Private religiosity	3.33 (1.76)	2.4 (1.79)	*
Internal religiosity	10.95 (3.91)	7.7 (4.38)	*
Events of discrimination	5.53 (2.24)	2.61 (1.16)	*
Frequency of discrimination	14.51 (8.18)	4.87 (4.21)	*
Anxiety	2.9 (3.52)	3.01 (3.64)	
Depression	3.34 (4.76)	5.37 (5.58)	*
General stress	4.47 (4.52)	5.09 (4.59)	
COVID stress	4.05 (2.83)	3.42 (2.55)	

One participant did not identify as male or female. Mean (SD); Significant = racial group difference; *p < 0.05. Sleep quality refers to the global score of the Pittsburgh Sleep Quality Index (PSQI). Sleep habits are the total score for the Sleep Hygiene Index (SHI). Sleep quality >5 indicates global PSQI scores greater than 5. We performed a chi-square test to assess racial differences in sleep quality scores >5 and those less than 5.

### 3.2 Positive and negative factors linked with sleep quality across age and racial group

Next, we assessed factors that were positively and negatively related to sleep quality using multiple linear regression analyses across age and racial group. For each positive and negative factor, we ran separate regression models, controlling for covariates, age, years of education, and education quality.

The positive factors included sleep habits, social support, and religiosity. Better sleep habits were significantly related to higher sleep quality [*B* = 0.262, *p* < 0.001, 95% CI: (0.207–0.316)]. Similarly, separate regression models revealed that greater positive social interaction and emotional social support were both linked with higher sleep quality [positive social interaction: *B* = −1.01, *p* < 0.001, 95% CI: (−1.450 to −0.572); emotional social support: *B* = −1.181, *p* < 0.001, 95% CI: (−1.656 to −0.706)]. There were no significant associations between religiosity and sleep quality (absolute *B’s* < 0.236, *p’s* > 0.079).

We also examined factors that may negatively impact sleep quality, including general stress, race-related stress, and COVID stress. For race-related and COVID stress, we controlled for the aforementioned covariates and general stress. Both general stress [*B* = 0.482, *p* < 0.001, 95% CI: (0.392–0.473)] and COVID stress [*B* = 0.194, *p* = 0.029, 95% CI: (0.020–0.368)] were significantly associated with poor sleep quality. There were no significant associations between race-related stress and poor sleep quality (absolute *B’s* < 0.64, *p’s* > 0.422).

### 3.3 Sleep quality more sensitive to COVID stress in White than Black adults

**FIGURE 1:**
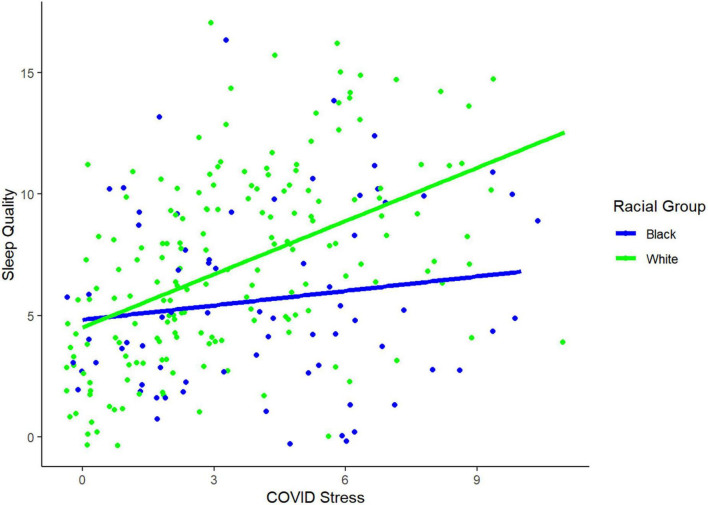
Racial group differences in association between poor sleep quality and greater COVID stress. The plot demonstrates that White adults show stronger associations between COVID stress and sleep quality than Black adults. Jitter was applied to this plot to better visualize the data.

We were interested in racial group differences in the link between lifestyle and psychosocial factors and sleep quality. Controlling for age, years of education, education quality, and general stress, moderation analyses revealed a significant interaction effect of racial/ethnic group X COVID stress on sleep quality [Δ*R2* = 0.02, *F*(1, 243) = 6.35, *p* = 0.012]. Follow-up partial correlations (controlling for the covariates) revealed that White adults were more sensitive to the negative effects of high COVID stress on sleep quality than Black adults were. In other words, higher COVID stress was associated with poorer sleep quality in White adults, but not Black adults [White: partial *r*(173) = 0.47, *p* < 0.001; Black: partial *r*(68) = 0.17, *p* = 0.152; see Figure 1]. There were no other significant racial group moderation effects (*p’s* > 0.149).

### 3.4 Protection against the negative effects of stress on sleep quality

**FIGURE 2:**
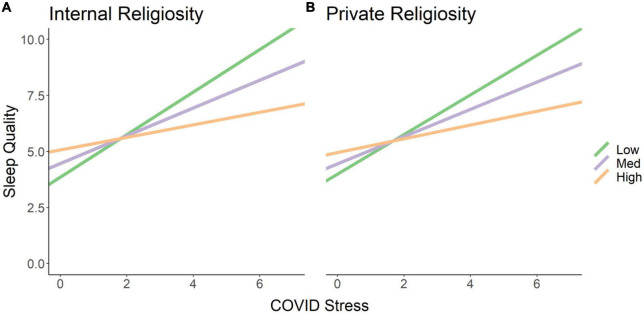
High religiosity protects against the negative effects of COVID stress on sleep quality. The simple slopes represent levels of religiosity: low (1 SD below the mean, green), med (mean, purple), and high (1 SD above the mean, orange). Higher endorsement of **(A)** internal and **(B)** private religiosity attenuates the negative association between high COVID stress and poor sleep quality.

Given the negative association between COVID stress and poor sleep and the racial group difference in the relationship between COVID stress and poor sleep, we were interested in if any positive factors protected against the negative effects of COVID stress on sleep quality. Therefore, we examined if social support or religiosity measures that did not depend on social activity (i.e., private, internal) moderated the association between COVID stress and sleep quality. Controlling for age, years of education, education quality, and general stress, moderation analyses demonstrated that those who were high on religiosity showed weaker associations between COVID stress and sleep quality than those who were low on religiosity [private religiosity: Δ*R2* = 0.03, *F*(1, 243) = 9.73, *p* = 0.002; see Figure 2A; internal religiosity: Δ*R2* = 0.02, *F*(1, 243) = 8.18, *p* < 0.005; see Figure 2B]. Thus, greater endorsement of religious activity blunted the relationship between high COVID stress and poor sleep quality. There were no significant moderation effects of social support (*p’s* > 0.832).

## 4 Discussion

We examined factors that protect against the negative effects of stress related to the COVID-19 pandemic in an adult lifespan sample of Black and White people. While previous research assessing sleep during COVID-19 has focused on sleep problems and associated negative factors (for a review, Alimoradi et al., 2021), we investigated positive factors that may protect against the negative effects of COVID-related stressors on sleep quality in Black and White adults. Here, we found that Black adults reported more formal education, greater religious activity, and higher quality sleep than White adults. We also found that sleep for White adults was more sensitive to the stress of COVID-19 than it was for Black adults. Specifically, White adults showed an association between higher COVID stress and poorer quality sleep, but no such relationship was found in the Black adults. Greater religiosity blunted the negative effects of COVID stress on sleep quality across racial groups. We discuss these results below.

In our sample, Black adults reported better sleep quality than White adults. Although this sleep result is inconsistent with the greater sleep literature on racial/ethnic sleep disparities (Chen et al., 2015; Johnson et al., 2019; Yip et al., 2021), there are several mechanisms that could influence this unexpected racial group difference in sleep quality in the present study. One is that Black adults reported greater religious activity than White adults during a time of high stress, namely, COVID-19. Black adults may have been able to better avoid stress-related reductions in sleep quality than White adults because of the protective effects of religiosity. Previous research has shown that Black adults use religious behaviors, particularly prayer, as a coping mechanism for racial discrimination (Hayward and Krause, 2015). The present results suggest that this coping strategy may extend to stress related to COVID-19. Another potential explanation for this unexpected result is that Black adults reported greater education than White adults in the present sample, and greater education has been linked with better sleep quality (Turner et al., 2016) and sleep habits (Nam et al., 2018). It should be noted, however, that Black adults do not typically report greater formal education than White participants (NCES, 2019). Given this racial difference in educational attainment in the present study, online data collection may be subject to selection bias and not representative of the general population. Moreover, participants in our sample reported poorer sleep quality than has been reported in studies before COVID-19 (e.g., Gamaldo et al., 2014). In any case, the present results demonstrate that racial sleep disparities are not always present and may be narrowed by protective factors, including high religious behaviors, as discussed below.

We found a protective effect of religious behaviors against the negative effects of COVID stress on sleep. Specifically, those who endorsed greater religious behaviors (e.g., prayer, meditation) did not show relationships between high COVID stress and poor sleep, while those who were low on religiosity did. Religiosity may act as a protective factor through several mechanisms. First, religiosity may directly reduce behaviors that may cause stress. For example, greater religious behaviors have been hypothesized to deter stress-inducing behaviors that are inconsistent with most religious ideologies, including dishonesty and criminality, that could influence poor sleep quality (for a review, Hill et al., 2018). Second, religious behaviors, especially meditation (Koenig and Büssing, 2010), have been linked with a greater sense of calm and emotion regulation. For example, several mindfulness-based meditation techniques have demonstrated reductions in stress following meditation training (for a review, Newberg, 2011). Thus, religious behaviors may be linked to experiencing less stress and being better able to deal with stress when it occurs, both of which could influence high quality sleep.

Unlike religious behaviors, social support did not buffer the negative effects of stress on sleep quality. While greater social support has been linked with lower pre-sleep arousal (e.g., worry or physical discomfort before bed) and higher quality sleep (Morin et al., 2003), we found no protective effects of social support in the present study. Notably, we collected the present data during the height of COVID-19 when physical distancing guidelines were in place, and this could have affected the perceptions and nature of social support. Future studies should hone in on both the positive and negative aspects of social support systems and their relative associations with sleep quality.

## 5 Strengths, limitations, and future directions

The present study has several strengths. This study addresses several critical gaps in the sleep literature by identifying positive factors that protect against the negative impact of stress on sleep quality in Black and White people across the adult lifespan during COVID-19. We examined race-related stressors that have been previously linked with poor sleep quality (for a review, Slopen et al., 2016). We are the first to assess religious activities in relation to sleep during COVID-19, and religiosity is often greater in Black adults than White adults (Taylor et al., 1996). The findings here will allow for a broader understanding of factors that protect against poor sleep quality in Black and White people. However, this study is not without limitations. We recruited participants using an online recruitment platform during COVID-19. Consequently, it is possible that our sample differed from existing studies in ways that affected the observed results. For example, we may have low numbers of Black people classified as essential workers in the present sample. Thus, the participants in our study may be experiencing lower levels of stress during COVID-19 than Black people recruited from a community-based sampling approach. The Black participants in our sample also reported higher educational attainment than non-Hispanic White adults, which is not typically found in large, epidemiological participant samples (Turner et al., 2016). These differences might have reduced our ability to detect poorer sleep quality in Black adults as compared to White adults, as is typically seen in the literature, particularly in community-based participant samples (Chen et al., 2015; Turner et al., 2016; Johnson et al., 2019). Moreover, there may be personality differences in those who volunteer for an online study as compared to those who do not. However, willingness to participate factors into selection bias in all studies and is unlikely to explain racial group differences in the findings presented here.

Our study is also limited by its cross-sectional design and only collecting self-reported sleep measures. Self-reported sleep measures are vulnerable to error, particularly regarding estimates of sleep duration and sleep continuity (King et al., 2017). Moreover, longitudinal assessments of sleep quality, especially before and after COVID-19, may provide better insight into racial differences in sleep quality that were found in the present study. For example, Black adults, protected by greater religious activity, may not have demonstrated a large decline in sleep quality from before the onset of the pandemic, relative to White adults. Although one longitudinal study has found better sleep quality in relation to quarantine during COVID-19 (Gao and Scullin, 2020), future research should assess racial differences for changes in sleep quality once COVID-19 is over. Gao and Scullin (2020) suggested that there may be a sleep benefit to the schedule flexibility of working from home. Future studies should determine if these sleep improvements are sustained across time. Future research should also employ objective sleep measurements such as actigraphy and polysomnography in addition to self-reported sleep quality. These multimodal measures of sleep quality would allow for a better representation of sleep health instead of only assessing sleep problems and sleep complaints (for a review, Buysse, 2014).

## 6 Conclusion

Black adults often experience poorer sleep quality as compared to White adults (for a review, Johnson et al., 2019). In the present sample, Black adults reported higher educational attainment and greater religiosity than White adults, and those positive factors may have facilitated high resilience to stress during COVID-19. We found that religiosity dulled the negative effects of stress from COVID-19 on sleep quality across racial groups. Our results suggest that racial sleep disparities may be narrowed by engaging in protective behaviors such as participating in private, religious activities. Considering the multitude of effects that sleep has on cognition and overall health (for a review, Buysse, 2014), future research should prioritize investigating factors that protect against experiencing poor sleep quality in racially/ethnically diverse people across the lifespan.

## Data availability statement

The raw data supporting the conclusions of this article will be made available by the authors, without undue reservation.

## Ethics statement

The studies involving human participants were reviewed and approved by Georgia Tech IRB. The patients/participants provided their written informed consent to participate in this study.

## Author contributions

EH conceptualized the study design and collected and analyzed the data. AA and JC helped to collect and analyze the data. AA designed the figures and table. AD guided the conceptualization of the study and the interpretation of the results. EH and AD wrote the manuscript. All authors contributed to the article and approved the submitted version.

## Footnotes

1
qualtrics.com
