# Supplementary material for: Factors that protect against poor sleep quality in an adult lifespan sample of non-Hispanic Black and non-Hispanic White adults during COVID-19: A cross-sectional study
Source: Front Psychol. 2022 Dec 15;13:949364. doi: 10.3389/fpsyg.2022.949364 (PMC9798098; doi:10.3389/fpsyg.2022.949364)
Supplement: Supplementary file 1 [file Data_Sheet_1.docx]

Supplementary Material

**Appendix A. Questionnaires**

**COVID Stress:** lab-developed

The following questions pertain to changes that you may have experienced related to COVID-19. Please rate on a scale of 0-3 how the following circumstances have affected you:

Response options included: Did not experience (0) Yes, but it did not bother me (1), Yes, it bothered me somewhat (2), Yes, it bothered me a lot.

1. A drop in income related to COVID- 19?
2. New family care duties (e.g., home schooling, child care, caring for an elderly parent, providing meals)?
3. Feelings of anxiety or depression?
4. Poor internet access at home?

**Sleep Quality and Duration:** Pittsburg Sleep Quality Index (Buysse et al., 1989; Tracy et al., 2021)

Response options for items 1-4 were write-in responses. Responses for items 5a-j and 6-8 included: Not during the past month (0), Less than once a week (1), Once or twice a week (2), Three or more times week (3). Responses for item 9 were: Very good (0), Fairly good (1), Fairly bad (2), Very bad (3).

1. When have you usually gone to bed? ______________
2. How long (in minutes) has it taken you to fall asleep each night? ______________
3. When have you usually gotten up in the morning? ______________
4. How many hours of actual sleep do you get at night? (This may be different than the number of hours you spend in bed) ______________
5. During the past month, how often have you had trouble sleeping because you...
6. Cannot get to sleep within 30 minutes
7. Wake up in the middle of the night or early morning
8. Have to get up to use the bathroom
9. Cannot breathe comfortably
10. Cough or snore loudly
11. Feel too cold
12. Feel too hot
13. Have bad dreams
14. Have pain
15. Other reason(s), please describe, including how often you have had trouble sleeping because of this reason(s):
16. During the past month, how often have you taken medicine (prescribed or “over the counter”) to help you sleep?
17. During the past month, how often have you had trouble staying awake while driving, eating meals, or engaging in social activity?
18. During the past month, how much of a problem has it been for you to keep up enthusiasm to get things done?
19. During the past month, how would you rate your sleep quality overall?

**Sleep Habits:** Sleep Hygiene Index (Chehri et al., 2022; Mastin et al., 2006)

Please indicate how frequently you engage in the following behaviors.

Response options included: Never (0), Rarely (1), Sometimes (2), Frequently (3), and Always (4).

1. I take daytime naps lasting two or more hours.
2. I go to bed at different times from day to day.
3. I get out of bed at different times from day to day.
4. I exercise to the point of sweating within 1 h of going to bed.
5. I stay in bed longer than I should two or three times a week.
6. I use alcohol, tobacco, or caffeine within 4 h of going to bed or after going to bed.
7. I do something that may wake me up before bedtime (for example: play video games, use the internet, or clean).
8. I go to bed feeling stressed, angry, upset, or nervous.
9. I use my bed for things other than sleeping or sex (for example: watch television, read, eat, or study).
10. I sleep on an uncomfortable bed (for example: poor mattress or pillow, too much or not enough blankets).
11. I sleep in an uncomfortable bedroom (for example: too bright, too stuffy, too hot, too cold, or too noisy).
12. I do important work before bedtime (for example: pay bills, schedule, or study).
13. I think, plan, or worry when I am in bed.

**Social Support:** (Salgado et al., 2022; Sherbourne & Stewart, 1991)

People sometimes look to others for companionship, assistance, or other types of support. How often is each of the following kinds of support available to you if you need it? Choose one number from each line.

Response options included: None of the time (1), A little of the time (2), Some of the time (3) Most of the time (4), All of the time (5).

**Emotional Support**

1. Someone you can count on to listen to you when you need to talk
2. Someone to give you good advice about a crisis
3. Someone to give you information to help you understand a situation
4. Someone to confide in or talk to about yourself or your problems
5. Someone whose advice you really want
6. Someone to share your most private worries and fears with
7. Someone to turn to for suggestions about how to deal with a personal problem
8. Someone who understands your problems

**Positive Social Interaction**

1. Someone to have a good time with
2. Someone to get together with for relaxation
3. Someone to do something enjoyable with

**Religiosity:** The Duke Religion Index (Koenig & Büssing, 2010; Thomas et al., 2018)

**Private Religiosity**

Response options included: Rarely or never (1), A few times a month (2), Once a week (3), Two or more times/week (4), Daily (5), More than once a day (6).

1. How often do you spend time in private religious activities, such as prayer, meditation or Bible study?

**Internal Religiosity**

The following section contains 3 statements about religious belief or experience. Please mark the extent to which each statement is true or not true for you.

Response options included: Definitely not true (1), Tends not to be true (2), Unsure (3), Tends to be true (4), Definitely true of me (5).

1. In my life, I experience the presence of the Divine (i.e., God)
2. My religious beliefs are what really lie behind my whole approach to life - (IR)
3. I try hard to carry my religion over into all other dealings in life - (IR)

**Depression, Anxiety, and Stress:** Depression, Anxiety, and Stress Scale-21 (Brown et al., 2022; Cavuoto et al., 2016; Lovibond & Lovibond, 1995)

Response options included: Did not apply to me at all (0), Applied to me to some degree or some of the time (1), Applied to me a considerable degree or a good part of the time (2), Applied to me very much or most of the time (3).

**Depression**

1. I couldn’t seem to experience any positive feeling at all
2. I found it difficult to work up the initiative to do things
3. I felt that I had nothing to look forward to
4. I felt down-hearted and blue
5. I was unable to become enthusiastic about anything
6. I felt I wasn’t worth much as a person
7. I felt that life was meaningless

**Anxiety**

1. I was aware of dryness of my mouth
2. I experienced breathing difficulty (e.g. excessively rapid breathing, breathlessness in the absence of physical exertion)
3. I experienced trembling (e.g. in the hands)
4. I was worried about situations in which I might panic and make a fool of myself
5. I felt I was close to panic
6. I was aware of the action of my heart in the absence of physical exertion (e.g. sense of heart rate increase, heart missing a beat)
7. I felt scared without any good reason I felt that life was meaningless

**Stress**

1. I found it hard to wind down
2. I tended to over-react to situations
3. I felt that I was using a lot of nervous energy
4. I found myself getting agitated
5. I found it difficult to relax
6. I was intolerant of anything that kept me from getting on with what I was doing
7. I felt that I was rather touchy

**Discrimination:** Events of Discrimination Scale (Held et al., 2022; Krieger et al., 2005)

Have you ever experienced discrimination, been prevented from doing something, or been hassled or made to feel inferior in any of the following situations because of your race, ethnicity, or color?

Response options included: no (0), once (1), two or three times (2.5), four or more times (5).

1. At school?
2. Getting hired or getting a job?
3. At work?
4. Getting housing?
5. Getting medical care?
6. Getting service in a store or restaurant?
7. Getting credit, bank loans, or a mortgage?
8. On the street or in a public setting?
9. From the police or in the courts?

**Appendix B. Supplemental Figure**

**
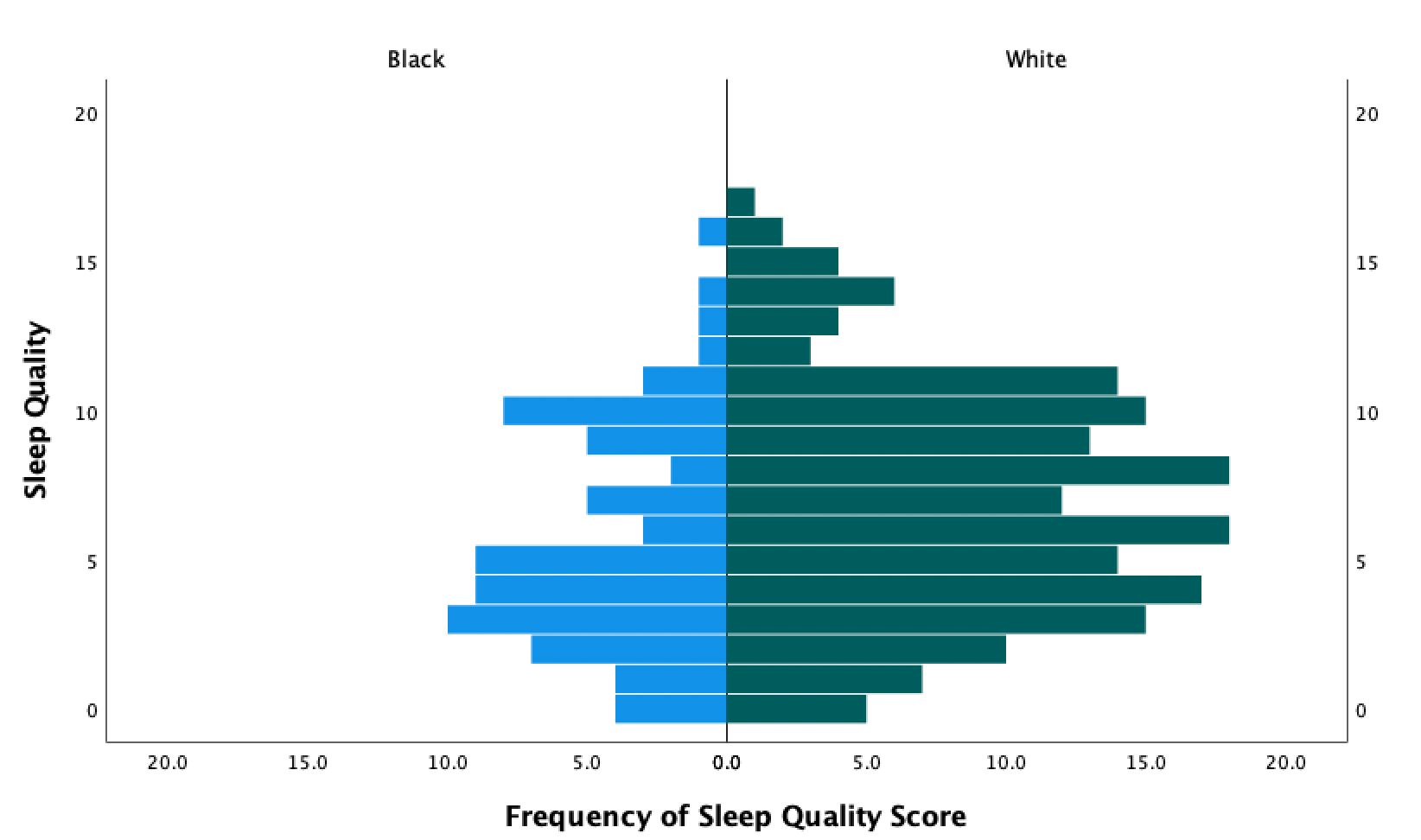
**

**Supplemental Figure 1.** Distribution of global sleep quality, measured with the PSQI, by racial group.

References

Brown, C. R. H., Feng, Y.-C., Costin, V., Hirsch, C. R., Wang, Y.-H., Wang, Y.-L., Chew, J., Kenny, J., & Allen, P. (2022). Specific Pandemic-Related Worries Predict Higher Attention-Related Errors and Negative Affect Independent of Trait Anxiety in UK-Based Students. *Cognitive Therapy and Research*, 1–19. https://doi.org/10.1007/s10608-022-10336-7

Buysse, D. J., Reynolds, C. F., Monk, T. H., Berman, S. R., & Kupfer, D. J. (1989). The Pittsburgh sleep quality index: A new instrument for psychiatric practice and research. *Psychiatry Research*, *28*(2), 193–213. https://doi.org/10.1016/0165-1781(89)90047-4

Cavuoto, M. G., Ong, B., Pike, K. E., Nicholas, C. L., Bei, B., & Kinsella, G. J. (2016). Objective but not subjective sleep predicts memory in community-dwelling older adults. *Journal of Sleep Research*, *25*(4), 475–485. https://doi.org/10.1111/jsr.12391

Chehri, A., Taheri, P., Khazaie, H., Jalali, A., Ahmadi, A., & Mohammadi, R. (2022). The relationship between parents’ sleep quality and sleep hygiene and preschool children’ sleep habits. *Sleep Science (Sao Paulo, Brazil)*, *15*(3), 272–278. https://doi.org/10.5935/1984-0063.20220051

Held, M. L., First, J. M., & Huslage, M. (2022). Effects of COVID-19, Discrimination, and Social Support on Latinx Adult Mental Health. *Journal of Immigrant and Minority Health*. https://doi.org/10.1007/s10903-022-01382-0

Koenig, H. G., & Büssing, A. (2010). The Duke University Religion Index (DUREL): A Five-Item Measure for Use in Epidemological Studies. *Religions*, *1*(1), Article 1. https://doi.org/10.3390/rel1010078

Krieger, N., Smith, K., Naishadham, D., Hartman, C., & Barbeau, E. M. (2005). Experiences of discrimination: Validity and reliability of a self-report measure for population health research on racism and health. *Social Science & Medicine*, *61*(7), 1576–1596. https://doi.org/10.1016/j.socscimed.2005.03.006

Lovibond, S. H., & Lovibond, P. F. (1995). *Manual for the Depression Anxiety Stress Scales* (2nd ed.). Psychology Foundation.

Mastin, D. F., Bryson, J., & Corwyn, R. (2006). Assessment of Sleep Hygiene Using the Sleep Hygiene Index. *Journal of Behavioral Medicine*, *29*(3), 223–227. https://doi.org/10.1007/s10865-006-9047-6

Salgado, S., González-Suhr, C., Nazar, G., Alcover, C.-M., Ramírez-Vielma, R., & Bustos, C. (2022). Relationships between Individual and Social Resources, Anxiety and Depression in the Early Lockdown Stage by the COVID-19 in Chile. *Behavioral Sciences (Basel, Switzerland)*, *12*(10), 357. https://doi.org/10.3390/bs12100357

Sherbourne, C. D., & Stewart, A. L. (1991). The MOS social support survey. *Social Science & Medicine*, *32*(6), 705–714. https://doi.org/10.1016/0277-9536(91)90150-B

Thomas, K. H., McDaniel, J. T., Albright, D. L., Fletcher, K. L., & Koenig, H. G. (2018). Spiritual Fitness for Military Veterans: A Curriculum Review and Impact Evaluation Using the Duke Religion Index (DUREL). *Journal of Religion and Health*, *57*(3), 1168–1178. https://doi.org/10.1007/s10943-018-0597-z

Tracy, E. L., Chin, B., Lehrer, H. M., Carroll, L. W., Buysse, D. J., & Hall, M. H. (2021). Coping strategies moderate the effect of perceived stress on sleep and health in older adults during the COVID-19 pandemic. *Stress and Health*, *n/a*(n/a), 1–14. https://doi.org/10.1002/smi.3124
